# Qualitative case study of needle exchange programs in the Central Appalachian region of the United States

**DOI:** 10.1371/journal.pone.0205466

**Published:** 2018-10-12

**Authors:** Stephen M. Davis, Danielle Davidov, Alfgeir L. Kristjansson, Keith Zullig, Adam Baus, Melanie Fisher

**Affiliations:** 1 Department of Health Policy, Management, and Leadership, West Virginia University, Morgantown, United States of America; 2 Department of Emergency Medicine, West Virginia University, Morgantown, United States of America; 3 Department of Social and Behavioral Sciences, West Virginia University, Morgantown, United States of America; 4 Department of Medicine, Section of Infectious Diseases, West Virginia University, Morgantown, United States of America; University of Arkansas for Medical Sciences, UNITED STATES

## Abstract

**Background:**

The Central Appalachian region of the United States is in the midst of a hepatitis C virus epidemic driven by injection of opioids, particularly heroin, with contaminated syringes. In response to this epidemic, several needle exchange programs (NEP) have opened to provide clean needles and other supplies and services to people who inject drugs (PWID). However, no studies have investigated the barriers and facilitators to implementing, operating, and expanding NEPs in less populous areas of the United States.

**Methods:**

This qualitative case study consisted of interviews with program directors, police chiefs, law enforcement members, and PWID affiliated with two NEPs in the rural state of West Virginia. Interview transcripts were coded inductively and analyzed using qualitative data analysis software. Final common themes related to barriers and facilitators of past program openings, current program operations, and future program plans, were derived through a consensus of two data coders.

**Results:**

Both NEPs struggled to find existing model programs, but benefited from broad community support that facilitated implementation. The largest operational barrier was the legal conundrum created by paraphernalia laws that criminalize syringe possession. However, both PWID and law enforcement appreciated the comprehensive services provided by these programs. Program location and transportation difficulties were additional noted barriers. Future program operations are threatened by funding shortages and bans, but necessitated by unexpected program demand.

**Conclusion:**

Despite broad community support, program operations are threatened by growing participant volumes, funding shortages, and the federal government’s prohibition on the use of funds to purchase needles. Paraphernalia laws create a legal conundrum in the form of criminal sanctions for the possession of needles, which may inadvertently promote needle sharing and disease transmission. Future studies should examine additional barriers to using clean needles provided by rural NEPs that may blunt the effectiveness of NEPs in preventing disease transmission.

## Introduction

Hepatitis C virus (HCV) is a blood-borne infection that is commonly transmitted during injection drug use [1, 2]. HCV affects an estimated 177.5 million of the world’s population [3], and is the leading cause of advanced liver diseases [4]. The worldwide economic burden associated with HCV-related liver disease is enormous and includes both direct costs related to medical care and indirect costs such as loss of work productivity [4, 5]. In the United States, estimated health care costs associated with the treatment and care of chronic HCV was $6.5 billion in 2011, and is expected to peak at $9.1 billion in 2024 [6].

Although most countries have seen rates of HCV plateau [7], incident cases of HCV infection increased more than 2.9 fold in the United States between 2010 and 2015 [8]. Many of these new infections have been observed in white, young, people who inject drugs (PWID) residing in nonurban areas of the United States [2, 8–12]. Between 2006 and 2012, the rural, central Appalachian region of the United States (Kentucky, Tennessee, Virginia, and West Virginia) observed a 364% increase in acute HCV cases in young persons (≤ 30 years old) [12]. There is evidence that this exponential increase is highly correlated with a regional prescription opioid misuse epidemic and an increase in injection of street drugs (i.e., drugs not legally obtained from a pharmacy or physician), particularly heroin, that occurred during the same time period [12–14].

The rural state of West Virginia, located entirely in the Appalachian region, has the second-highest rate of incident cases of HCV in the country (3.4 per 100,000)[8] and shares many of the demographic characteristics of PWID who are thought to be driving the US epidemic (i.e., white and young). Between 2007 and 2015, the HCV incidence rate increased over 300% in West Virginia, and the most frequently reported risk factors for developing acute HCV infection were *injection drug use* and *used street drugs* [15]. In response this epidemic, needle exchange programs (NEPs) began opening in the state during 2015.

Despite the clear need for NEPs in rural states like West Virginia, there remains a dearth of research documenting the unique context and challenges to operating NEPs in rural areas of the United States that can provide critical information on the barriers and facilitators that impact overall program effectiveness [16]. A mail/telephone survey with the directors of NEPs operating in the United States in 2013 revealed that rural programs were more likely to report experiencing a lack of resources/funding compared to suburban and urban programs (73% versus 64% and 63%, respectively)[17]. However, challenges and barriers related to program design, implementation, and sustainability were not reported. Therefore, the purpose of this study was to conduct a qualitative case study of two NEPs that opened in West Virginia in 2015. As part of this case study, we compared and contrasted the experiences and contexts of each program to identify common themes related to program operations. Of specific interest were the facilitators and barriers to the implementation and ongoing operations of each program. In particular, this case study sought to answer the following questions: (1) What were the facilitators and barriers to opening the program in the past?; (2) What were the facilitators and barriers encountered during the day-to-day running of the program in the present?; (3) What are the future plans for the program?; and, (4) What are the anticipated challenges and barriers related to these future plans?

## Methods

### Approach

A multiple, intrinsic case study approach was selected for the following reasons: 1) each case (NEP) was of interest in and of itself (i.e., intrinsic)[18]; 2) the goal of this study was to obtain an in-depth understanding of each case[19]; and, 3) each program (case) was a bounded system[19]. For the purposes of this study, we bounded each case to the specific program, and examined the past, present, and future time orientations of each program.

### Sampling

Each case was selected using purposive and snowball sampling strategies [20]. West Virginia currently has 10 NEPs registered with the North American Syringe Exchange Network (NASEN)[21]. For this study, we purposefully selected two of the longest operating programs, a NEP operating within a free healthcare clinic and a NEP operating within a health department, to glean important insights from their significant experiences. This sample size is consistent with current qualitative case study sample size recommendations of no more than 5 cases [19]. We initially interviewed the two NEP program directors and then subsequently interviewed law enforcement officials and NEP participants at each site as suggested by the directors (i.e. snowball sampling) to triangulate emergent themes and achieve data saturation, as recommended [19]. More specifically, triangulation in qualitative research is a validation strategy that makes use of multiple and different sources to corroborate evidence. In our purposive, snowball sampling scheme, we sought to validate the issues and themes raised in our initial data collection with program directors with other groups that had a primary stake in the needle exchange programs [19]. The NEP program directors facilitated access to police chiefs and exchange attendees. Each PWID participant received a $25 gift card to a local convenience store. The only requirement for participation was being at least 18 years of age.

### Data collection

To answer the central research questions, the lead author (SD) conducted in-depth interviews with the director of each program. At one site, other administrative personnel (i.e., the nursing director and site administrator) also participated in the interview. In-depth interviews were selected to obtain a deeper understanding of these novel programs. Director interviews occurred in March 2016, law enforcement interviews occurred during the summer of 2016, and interviews with program participants occurred during the summer of 2017.

Prior to conducting each interview, a semi-structured interview guide was developed. The structure of the interview guide was focused on the facilitators and barriers encountered during the past program implementation, present ongoing operations, as well as those anticipated to occur in the future. However, due to the interest in obtaining an in-depth understanding of the central research questions *from the viewpoint of respondents*, each interview was semi-structured, and conversations were allowed to emerge and flow in naturalistic directions that may have departed from the interview guide. Importantly, the specific questions included in the interview guide were also guided by initial discussions with each director and included questions of interest to him or her. Finally, a case study of the first NEP in the United States, Point Defiance [22], was also used as a source of potential questions.

Prior to starting the interview, interviewees were informed that the discussion was voluntary and could be ended at any time without penalty. Interviewees were also informed that all discussions were confidential and that the specific program would not be identified in any publications or presentations. Therefore, each case has been assigned a pseudonym (Free Clinic NEP and Health Department NEP).

Each interview took place in a private conference room or via telephone and lasted approximately 60 minutes. Interviews were recorded using a stereo handheld digital voice recorder [23]. As a backup measure, interviews were also recorded using a smartphone voice recorder application [24]. At various points throughout each interview, the interviewer summarized the discussion and sought clarification from the interviewee.

### Data analysis

Interviews were transcribed by a professional and entered into qualitative data analysis software for analysis [25]. Prior to formal analysis, the primary author (SD) read each transcript several times and made notes regarding initial codes and categories. Next, line by line, open coding was conducted iteratively on all transcripts by the primary author with the objective of inductively identifying approximately 5 to 7 general themes in the interest of parsimony. The initial codebook had *a priori* codes for “past”, “present”, and “future”, into which information on “barriers” and “facilitators” corresponding to implementing and opening the program (past time orientation), ongoing program operations (present time orientation), and program growth and expansion (future time orientation) was categorized. Beyond the general descriptions, no other definitions or codes were established a priori. Interpretations were also guided by the primary author’s experience with individuals suffering from addiction and social work training, which embraces the harm reduction model. As suggested by Yin, a cross case synthesis was conducted to denote similarities, differences, and emerging themes *between* the two cases [26].

After initial coding and classification of all transcripts by the primary author, intensive group discussion with a second coder with expertise in qualitative research (DD) with a goal of simple consensus was employed to finalize the categories and overall themes [27–29]. Prior to this intensive group discussion, the second coder independently read all transcripts and made notes regarding codes, categories, and themes for comparison to the initial analysis performed by the primary author. This approach is recommended by several methodologists in order to preserve the interpretive process at the core of qualitative analysis [30], and is consistent with current case study practice [31], in specific, and generally accepted qualitative research reporting criteria [32]. Themes were classified according to time orientation (i.e., past, present, future) and whether it reflected a barrier or facilitator to program operations. Respondent quotes that captured the essence of each theme were selected as the primary data outcomes. Findings and interpretations of the data were shared with interviewees (i.e., member checking) to assess the credibility of the interpretations, and naturalistic generalizations were made by comparing emergent themes with previously published literature [19]. This protocol was approved by West Virginia University’s Institutional Review Board.

## Findings

### Sample characteristics

Qualitative interviews were conducted with two program directors, a program administrator, a program nurse, two chiefs of police, two law enforcement officers, and eight PWID (4 male, 4 female) who attended the two programs (3 Free Clinic NEP attendees, 5 Health Department NEP attendees).

### Description of cases

#### Free Clinic NEP

The Free Clinic was founded by a group of concerned citizens on the premise that healthcare is a universal right that should be available to all citizens regardless of ability to pay. In response to an ever-growing need, the clinic has moved several times, occupying larger quarters each time, and currently occupies a two-story building with ten exam rooms, two waiting rooms, a medication room, conference rooms, offices, and storage areas. The agency has a staff of 21, a volunteer corps of more than 200, and provides more than 28,000 patient encounters and dispenses free medications in the millions to qualified patients every year. In addition to medication assistance, the Free Clinic provides primary health care with a professional staff of physicians, nurse practitioners, physician assistants, registered nurses (RNs), licensed practical nurses (LPNs), medical assistants, social workers, and therapists. Specialty clinics for various diseases (e.g., diabetes) and topics (e.g., women’s health) are also offered. Some dental care and mental health services are provided on a limited basis. All health care, including prevention, health awareness, and chronic disease management, are free of charge to qualified patients and are offered in clinics or in group education.

#### Precipitating event/program impetus

Although Free Clinic personnel were aware that some patients were also PWID, the significant incidence of HCV among this population was not discovered until the Centers for Disease Control (CDC) updated its HCV screening guidelines to recommend testing for all persons between 45 and 64 years of age [33]. The Free Clinic began implementation of the updated guidelines in June 2013, and within one month, slightly over 10% of 300 patients tested positive for HCV. This event led to an examination of factors related to the high HCV rate, which revealed that most of the HCV positive individuals either had a history of injection drug use or were currently injecting. Around the same time, clinic personnel became aware of a heroin epidemic in West Virginia that emerged from an opioid misuse epidemic [13]. Collectively, these two factors led to the further exploration of a NEP to prevent new HCV infections. Between July 2013 and October 2014, the Free Clinic conducted in-depth research regarding the startup and implementation of NEPs, and presented this information to the Board of Directors at the October meeting in 2014. The board unanimously approved the implementation of a NEP, which led to a quest to secure funding and logistical planning related to implementation. After obtaining support from the board to open a NEP, the Free Clinic NEP established a Community Advisory Board (CAB) comprised of members of the community, a representative of the police department, and a social worker. The CAB also sought information from current users to guide the decision-making process. The Free Clinic NEP served two clients the first day.

#### Program operations

The Free Clinic NEP is open one day each week for less than 5 hours, and makes special arrangements for participants who cannot attend during regularly scheduled hours. No other clinic services are provided during this time period. There are three staff members that are dedicated to the NEP, and students from a nearby university also assist with operations. PWID of all ages are accepted with a very specialized protocol for those under 18 years of age, although no children had yet been seen at the time of the interview. The average age of participants is 33 (range 19–70) with slightly more males than females (60% versus 40%, respectively). All but 3% of participants are White. Although West Virginia is a rural state, the Free Clinic NEP is located in a county not designated as rural by the U.S. Census. However, the overall population size of the entire county is less than 1/6^th^ the size of larger cities such as Baltimore, Maryland and Seattle, Washington where many of the first NEPs in the United States opened. Additionally, the Free Clinic NEP serves attendees that travel from surrounding census designated rural counties. Each participant is assigned a unique identification number for tracking purposes during the initial intake. Basic information regarding injection history, including duration and drug of choice, is also collected at this time. Clients are queried regarding whether or not they know their human immunodeficiency virus (HIV) and HCV status, and testing is encouraged. Importantly, all data collected in the context of the NEP is kept entirely separate from clinic medical records. Although not a requirement for participation, clients are encouraged to bring back used needles in a biohazard receptacle given to them by the program. The number of syringes given is based on client need. Need is determined by frequency of reported injections and transportation barriers (i.e., some attendees cannot attend every week due to long transportation times). There is no cap on syringes, and the program operates year-round. At the time of data collection, 200 unduplicated patients had attended the exchange since its opening.

Each participant leaves with approximately 30 to 50 clean needles. Additional drug paraphernalia, including cookers, cotton balls, alcohol swabs, and tourniquets, are also provided. The exchange offers rapid HCV testing as well as serum HIV testing. Participants also have access to a social worker and a nurse practitioner to discuss any health-related issues. Discussions regarding harm reduction, healthcare, treatment, housing, and general case management are common.

#### Health Department NEP

The Health Department NEP is located within a public health agency that provides a plethora of environmental and epidemiological public health services including environmental services (e.g., food safety, etc.), clinic services (e.g., sexually transmitted infection (STI) clinics, immunizations, etc.), threat preparedness, and disease investigation. The Health Department NEP is located in a county similar in size to the Free Clinic NEP, and also serves residents from surrounding rural counties.

#### Precipitating event/program impetus

In November 2014, a local Office of Drug Control Policy was created by the mayor of the town in which the health department is located. An investigation by this office into the problem of drug use found an extremely high rate of drug overdose, primarily from heroin. However, most concerning was the observed rising death rate. During this same time period in 2015, Scott County, Indiana was in the midst of an HIV epidemic from injection of illegal drugs with contaminated needles that led to the opening of a NEP to combat the epidemic [34]. These two factors led to the decision to explore the implementation of a NEP in this local West Virginia community. After extensive research on other NEPs (including visits to a small rural community in a neighboring state in early 2015), obtaining legal clearance, and the acquisition of adequate funding, the Health Department NEP formally opened. At the time of data collection, the NEP had seen 977 unique clients and exchanged 2,500 needles since its opening.

#### Program operations

The Health Department NEP runs three exchange rooms for at least five hours, one day each week. In contrast to the Free Health Clinic NEP, other health department services are also offered during the same time period. Approximately 15 individuals comprised of seven staff members, pharmacy, nursing, and medical students, and recovery coaches are present during operating hours. Approximately 130 participants use the exchange each week, with a single day high-volume of 144 participants observed. Participants receive a maximum of 40 clean needles, and most participants bring in approximately 30 to 40 used needles to exchange. In addition to exchanging needles, the Health Department NEP provides recovery coaches, STI testing, as well as testing for HCV (serum) and HIV. Participants are also queried regarding their infectious disease status, and are provided access to nurses who can examine their injection sites for potential infection. Teaching regarding safe and clean injection is also provided, and participants with infected sites are seen by a nurse practitioner. The administrative staff described difficulties related to the fact that routine health department services are offered during the times that the NEP is open. For example, many of the NEP participants were smokers who were frequently going outside to smoke, which disrupted other health department clientele.

Participants of the Health Department NEP are heterogeneous and contain a range of individuals from PWID who are gainfully employed despite their addictions to one or more substances to those that are unemployed and/or homeless. Although identification is not required by the Health Department NEP, the created ID number assigned to each participant for tracking purposes includes birth year. Gender is split evenly, and 96% of participants are White, which is similar to West Virginia as a whole (94% White) [35]. The average age is 37 and ranges from 21 to the 60.

### Emergent themes

Overall, the iterative, open coding process yielded 7 general themes: 2 themes related to program implementation and opening (PAST), 4 themes related to ongoing program operations (PRESENT), and 1 theme related to future growth and expansion (FUTURE).

#### 1. One Size Doesn’t Fit All (Barrier)

The theme, *One Size Doesn’t Fit All*, was identified from NEP directors’ descriptions of difficulties (i.e., barriers) encountered with locating an existing exchange program to serve as a model for the development of a NEP in their rural communities (see Table 1).

**Table 1:** PAST (Program implementation and opening) themes.

Theme	Category	Classification	Illustrative Quotes
**1. One Size Doesn’t Fit All**	a. Lack of a model program for smaller communities	Barrier	*So we did a lot of research and we found out that in some cities like Baltimore there’s a syringe exchange on almost every corner at different times during the week*. *But we didn’t find any information about small town USA*. *And I’ve also been involved in outreach in [another rural community] and have just recently did a conference call with several smaller communities*. *That’s the key here*. *The smaller communities are trying to break into this but the only model that’s out there is really for more urban areas*. *(Free Clinic NEP Director)*
**2. Like A Good Neighbor**	a. Community support	Facilitator	*So we did a very quiet start*. *We did not publicize*..... *we knew that this would generate emotional feelings and thoughts and that we hoped people would call and ask questions and try their best to understand*. *The response was 14*,*000 dollars*, *and we did not ask for money*. *(Free Clinic NEP Director)* *… [A] community meeting happened in March through a local*, *I don’t know what you call her*, *just a local community member that was interested and 500 people attended that*.....*This was organized as a Facebook campaign by a community person*. *This was not organized by any official*. *They got the room at the library and there were 500 people up there and it was testimony after testimony from families who had lost people from people who are recovered from this addiction*. *And there was uniform support*. *And in fact it went beyond the uniform support for the effort to the question*, *“Why are you not doing this*?*” (Health Department NEP Administrator)*

The Free Clinic NEP enlisted the support of students from a nearby university to conduct research on the existence of comparable exchange programs to no avail. This absence of model programs led to the need to modify elements of existing program policies from states such as New York to include policies for dealing with underage participants. Similarly, although the Health Department NEP was able to visit a program that was implemented in a nearby small community of approximately 5,000 residents, this program only exchanged needles and provided pamphlets with information, as opposed to the more comprehensive program models that include a variety of health services offered in other, more urban settings. Some NEP policies and procedures information was located on the Web, but the Health Department did not have the luxury of time to refine policies and procedures prior to program operations due to exponential program demand.

#### 2. Like a Good Neighbor (facilitator)

The theme, *Like a Good Neighbor*, included participant descriptions of the facilitative role that support from the community played in program implementation. Despite difficulties related to finding model programs during program development, both program openings were significantly facilitated by support from the community, albeit in somewhat different ways (see Table 1). The Free Clinic was initially concerned about a potential absence of community support and choose a silent opening that did not involve media announcements. However, once such announcements were made, the result was an overwhelming level of support. In contrast, the Health Department NEP enjoyed broad community support due to concern over the overwhelming number of opioid overdose deaths.

Present Barriers and Facilitators to Operating the Program

#### 3. The Legal Conundrum (major barrier)

The theme, *The Legal Conundrum*, was identified from participant discussions of the significant impact of paraphernalia laws and policing behaviors on program operations and participation (see Table 2). Both programs cited the critical importance of law enforcement support to successful program implementation and operations. However, existing paraphernalia laws and the fact that injection of illicit substances is an illegal activity created a quandary for law enforcement officials regarding the best approach to the possession of drug paraphernalia that may threaten the harm reduction goal of the NEPs. This quandary created confusion among both law enforcement and program participants. Although law enforcement leadership provided significant, crucial support for the programs and proffered directives to not seize clean needles in certain circumstances, variations in the particular actions taken in the case of clean (unused) needles were recorded between law enforcement leadership and staff. Some officers felt that giving citations for syringes would be a mechanism for linking participants to treatment via a law enforcement diversion program whereby participants would be sent to court-ordered treatment instead of jail. It is therefore not surprising that participants reported receiving legal sanctions for having clean needles obtained at the exchange despite showing proof (i.e., NEP identification card) when being stopped or searched for other reasons. These experiences created confusion among NEP participants who believed that clean needles obtained from the exchange could be possessed, albeit in somewhat limited circumstances.

**Table 2:** PRESENT (ongoing program operations) themes.

Theme	Categories	Classification	Illustrative Quotes
**3. The Legal Conundrum**	a.Paraphernalia laws that lead to confiscation and/or arrest of PWID for possessing needles	Barrier	*So the police officer who participates got permission from the chief of police to participate and then we*, *our medical director and myself went over and had a face to face meeting with our city police chief because we felt like we needed them on our side*..* *.* *.*we didn’t want to get to a point where we were giving out syringes*, *they would arrest somebody*, *confiscate those syringes and then*… *basically harm reduction is now no longer available to the community*. *(Free Clinic NEP Director)*
	b. Law enforcement confusion regarding the action to take upon discovery of needles c. Differing opinions between law enforcement leadership and staff	Barrier	*What my instructions have been is if you catch somebody coming out of [the NEP] with a bag of clean needles*, *or somebody that comes out of pharmacy*, *that’s not really drug paraphernalia*. *It’s got to be in conjunction with you know you’ve got the spoon*, *you’ve got the tie off*, *you know it’s clear that that’s what they’re using it for*. *And that’s typically what*, *you know we’re going on overdose calls*, *we’ll make an arrest in finding paraphernalia in you know the rig as they call it on somebody and charge them that way*. *(White*, *Male*, *Police Chief)* *It’s still drug paraphernalia…If you’re stopped by law enforcement*, *it’s not an exemption because you’re not supposed to be out here walking the streets carrying your drug paraphernalia and that kind of stuff*. *You know like I said they know what they’re dealing with and they know the actual laws for the use of*. *Yeah*, *don’t draw attention to yourself and then you might get by*. *(White*, *Male*, *Police Officer)*
	d. PWID reports of needle confiscation and/or legal sanctions for possession of needles whether clean or used	Barrier	*I had to go to court*. *I had three of them*. *They weren't even used*. *They were brand new*. *I got a $195*.*00 fine*. *(White*, *Female*, *PWID*, *Health Department NEP Participant)* *I went to court yesterday over paraphernalia charges because I was caught with three rigs*…*They were clean*…*Brand new*… *I got $175 for each needle*. *(White*, *Male*, *PWID*, *Health Department NEP Participant)* *Right here in this town*, *I got a misdemeanor possession*, *15 grams or less*. *How retarded is that*? *15 grams or less*? *I could see if it was dirty*, *and I didn't have no lids for it*, *you know what I mean*? *(White*, *Male*, *PWID*, *Free Clinic NEP Participant)*
	e. PWID confusion over whether or not needles obtained from the exchange can be possessed	Barrier	*They give you a card and I got pulled over and I showed them that card and they said that*, *that didn't matter at all*. *All that did was show where I got them from*, *that it was still paraphernalia and that it was still a misdemeanor*. *(White*, *Female*, *PWID*, *Health Department NEP Participant)* *I came across a policeman*, *and he was searching my stuff because I was around someone on house arrest*. *He was into my stuff*. *He seen my card from the [NEP]*. *He was like*, *"This is illegal*. *I know why you go there*. *This is illegal*.*" I'm like*, *"No*, *it's not*. *It's not illegal to go there and get syringes*.*" He's like*, *"Yes*, *it is*, *and I'm going to make sure they close down”*. *(White*, *Female*, *PWID*, *Free Clinic Participant)*
**4. Location is Everything**	a. Program location unreachable by some participants in the rural setting	Barrier	*Yeah [PWID] will car pool*. *They'll put five*, *six people in a car to come down here and to do the needle exchange*..* *.* *.*it's like now*. *It's been months since we've been able to get down here and do this*. *Today is the first day*. *(White*, *Female*, *PWID*, *Health Department NEP Participant)* …*[you can get to the exchange] if you have money for public transportation*, *stuff like that*, *but normally that's how you buy your drugs*. *You don't have a dollar on you*. *(White*, *Female*, *PWID*, *Health Department NEP Participant)* *I have a friend in PA*, *and they don't do anything like this*. *She asks me all the time like*, *"I wish I lived in West Virginia*, *so I could get them*.*” (White*, *Female*, *PWID*, *Free Clinic NEP Participant)*
	b. Program located near police department	Barrier	*It absolutely sucks*…*on one side of the street you go to jail and the other side of the street you go to get your needles so you can continue on with illicit behavior*. *It’s a mixed message*. *It’s a mixed metaphor*. *(White*, *Male*, *Police Chief)*
**5. Harm Reduction For All**	a. Potential to reduce the spread of disease for program participants b. Potential to reduce the spread of disease for law enforcement officials	Facilitator Facilitator	*It's a life changer*, *really*. *It's a life changer that you can come up here once or twice a week and get what you need other than going and trying to go and find it [needles] on the street*. *It's saving lives*. *(White*, *Female*, *PWID*, *Free Clinic*, *Participant)* *Well myself personally you know I’ve got a family*. *You know I don’t want to be out here and you know being on the front line dealing with these addicts you know*. *I don’t want to be stuck with a dirty needle*. *I’ve been stuck with a dirty needle before and it’s not very pleasant the treatment that you have to go through*. *(White*, *Male*, *Police Officer)*
	c. Concerns over increasing amounts of discarded needles in the community	Barrier	*I took my kid to the park*, *and I'm not kidding you*, *the only thing I don't like about it [the exchange]*, *and it's people in general*, *some people* … *I take my child to the park*, *and you see something like that [needle] laying on the ground*. *It's my child*. *What I do to myself is on me*, *and that's something I have to live with*. *I'm not going to do it in front of my child*. *To me that's disrespectful*. *Getting rid of them on the street*. *They was thinking about stop doing it [the exchange] because people was throwing them out like that*. *(White*, *Female*, *PWID*, *Free Clinic NEP Attendee)* …*when you do this program that this is a secondary effect because there are people who see them in the community parks*, *they see them in the playgrounds*. *They get thrown in trashcans and your sanitation workers in the city grab these bags and throw them in and they’re going to get needle sticks*. *(Health Department NEP Administrator)*
**6. Not Just A Needle**	a. Comprehensive harm reduction services beyond just the provision of needles	Facilitator	*You get everything that you need to use*. *They even give you something that you can throw them away in instead of just tossing them*. *(White*, *Female*, *PWID*, *Free Clinic NEP)* *Helps you stay sanitary*. *They tell you how to use everything all that*, *give you instructions and all that*. *(White*, *Male*, *PWID*, *Health Department NEP Participant)*
	b. Linkage to drug treatment services made the program more acceptable to law enforcement members	Facilitator	*But then you also have that real fine line*. *Are you encouraging the continued use of an illegal controlled substance or are you treating it*? *And I think the panacea is there are two different things*. *You’re trying to intervene on the [blood borne] pathogens*. *And through that blood borne pathogen intervention you’re trying to reach out to change the thought processes of the illicit drug user*. *And I think that’s where people lose the message here that it’s not one you know feeding the other*. *It’s one or the other*, *because that’s what you have*. *The needle exchange is a blood born pathogen intervention not a drug intervention*. *But they’re trying to pull the drug intervention in by getting a hold of them through the medical intervention*. *(White*, *Male*, *Police Chief)* *We probably wouldn’t have bought into it*, *wouldn’t have supported it had there not been a detox part of it*. *I know that public funding probably would not have been there had there not been a process of detox*, *not just here*, *here’s your needle*. *(White*, *Male*, *Police Chief)*

#### 4. Location Is Everything (barrier)

The theme, *Location Is Everything*, was identified from participant discussions of difficulties related to program utilization stemming from the physical location of the exchanges (see Table 2). One NEP was located very near to the police department, which was acknowledged by all parties interviewed to *“absolutely suck*.*”* Participants in the surrounding community perceived the other NEP as being too far away to promote regular attendance. The Free Clinic NEP had some participants that traveled from a rural county that is a two-hour drive one way due to the absence of such programs in the local community, and the Health Department NEP expressed an interest in expanding to an adjacent area where the death rate is high but distance precludes attendance by some users. One participant mentioned driving one hour, one way to obtain injection equipment, which precluded frequent and regular exchange attendance. All participants agreed on the need for expanded NEP services at other locations to reach more users. At the time of this writing, the Free Clinic NEP obtained funding to purchase a van for a mobile NEP, and several other NEPs have opened throughout West Virginia with plans for additional programs pending.

#### 5. Harm Reduction for All (barrier and facilitator)

The theme, *Harm Reduction for All*, included discussions of how the NEP could positively reduce the spread of disease for all participants (i.e., law enforcement, participants, and the surrounding community) (see Table 2). Law enforcement support for NEPs was facilitated when the intervention was viewed as an effort to reduce harmful blood borne pathogens that could be contracted by a needle stick occurring during subject searches, and NEP attendance was facilitated by participants’ concern with the prevention of abscesses and other blood-borne infections, especially HIV. Paradoxically, the potential of removing used needles from the community via the exchange process was threatened by an increase in discarded needles in the community that represented a barrier to ongoing program operations (i.e., threats of shutting down the program due to discarded needles in the community). This situation led to discussions among both program directors and participants regarding strategies to promote safer needle disposal. In particular, participants requested more biohazard disposal kits and one program developed a response team to collect discarded needles from the community.

#### 6. Not Just A Needle (facilitator)

The theme, *Not Just a Needle*, included instances where participants described the importance of other supplies and services in addition to clean needles provided by the exchanges (see Table 2). Participants appreciated the other comprehensive services provided, such as infectious disease testing and medical care, and linkage to drug treatment was a very important facilitator of program acceptance among law enforcement.

Future barriers and facilitators to expanding program operations

#### 7. Supply and Demand (barrier)

The final theme, *Supply and Demand*, was identified from discussions of unexpected demand for exchange services that created additional barriers to program expansion and growth due to funding challenges (see Table 3). The Free Clinic NEP would like to expand services to more rural counties via a mobile van that was recently purchased; whereas, the Health Department NEP has plans to expand from the current single site to four sites. However, although the federal ban on NEP funding was recently lifted, funds cannot be used to purchase needles. Therefore, both programs have to rely on other sources to fund the syringes. The Free Clinic NEP primarily uses money from foundation grants and donors to pay for syringes and other paraphernalia; whereas, the Health Department NEP relied on donations from a local pharmacy of between 13,000 and 15,000 syringes, and another large donor who contributed 60,000 syringes. For both programs, this barrier limits the number of syringes that can be distributed, which can negatively impact the secondary exchange of syringes that occurs when an participant takes clean needles and distributes them to other PWID in the community.

**Table 3:** FUTURE (program growth and expansion) themes.

Theme	Categories	Classification	Illustrative Quotes
**7. Supply and Demand**	a. Funding shortages exacerbated by the federal government’s prohibition on using funds to purchase needles (SUPPLY)	Barrier	..* *.* *.*the more clean syringes that are out there the better*. *We don’t make the person come themselves*. *There are people who come in to pick up for other people*. *Other people are working or can’t get here so we do encourage that*. *But we do limit it to 40 because we don’t have an unlimited supply of syringes*. *We don’t have unlimited resources in that area*. *(Health Department NEP Director)* *We can see that we need to expand this*, *that we are overwhelmed by the volume of patients that we’re seeing*..* *.* *.*But we don’t have funding to go on with that*. *And we don’t have sustaining funding beyond the first year when you get right down to it*. *(Health Department NEP Director)* *At the time the federal government would not allow funding for syringe exchange and we felt hesitant to ask our donors to right off the bat start donating to that*. *So we found two*, *well we found one basically*, *one foundation that was willing to give us money and we got the letter that we were confirmed the day before we started the syringe exchange*. *We had intended to start no matter what*. *(Free Clinic NEP Director)*
	b. Unanticipated and overwhelming numbers of program participants (DEMAND)	Barrier	*Well we anticipated only having 75 clients in the first year*, *which was my naïve thinking I guess*, *our belief that the situation wasn’t as bad as it really is*. *And it was also based on thinking that most of these folks would be people we knew*. *(Free Clinic NEP Director)* *We initially planned for about 500 participants for a year*. *So September 16 [2016] we expected to be at 500*. *We were at 500 in about nine weeks*. *So we immediately knew that we made a small error in judgment*. *(Health Department NEP Director)*

## Discussion

This case study of two needle exchange programs that opened in West Virginia in 2015 reveals common barriers and facilitators that impacted program implementation, operations, and future planning. At the outset, both programs struggled to find existing programs to effectively serve as a model. Although each program had slightly different precipitating events that drove the need for such programs in each community, both observed attendance rates far greater than anticipated, and have faced significant funding challenges that impacts future expansion needs driven by increasing participant volumes and significant transportation barriers to program attendance. The legal conundrum created by legal distribution of clean needles in the presence of paraphernalia laws rooted in a criminal (i.e., moral) approach to drug use that impacts policing behaviors was a significant barrier to program operations. Despite these challenges, robust community support and the creative leveraging of volunteer resources have facilitated the successful implementation and operation of these programs.

In-depth case studies describing the development and implementation of NEPs in the United States are scant. The literature largely consists of brief descriptions of the first NEPs to open in large urban areas of the United States (e.g., Tacoma, San Francisco, New York City, Washington, DC, etc.) in the late 1980s and early 1990s in response to the HIV crisis [22, 36–42]. Only three studies [22, 40, 43] were located that specifically described the use of qualitative research methodologies to obtain an in-depth program (i.e., case) understanding. However, these differences notwithstanding, there were a number of similarities with regard to barriers and facilitators experienced by these urban programs and the two West Virginia exchanges that comprise this case study.

First, as was experienced by both West Virginia programs, there was no readily available model exchange in the United States to guide program design and implementation (*One Size Doesn’t Fit All theme*). Rather, many urban programs used the first NEPs that opened in Amsterdam as a guide to program development [22, 37–39]. Other programs engaged the surrounding community during program design and implementation [39, 40], and the NASEN has served as a mechanism to exchange lessons learned among developing NEPs. For example, the *Lifepoint* NEP case study described the use of qualitative ethnographic methods and systemic community analysis in the design and implementation of a NEP in Milwaukee, Wisconsin [40], which is similar to the community advisory board and key informants used to guide the development of the Free Clinic NEP. Program planners also engaged community leaders and law enforcement in the design and implementation of a NEP in New Haven, Connecticut [39].

Community support was cited as critical to program implementation and continuing operations by a few studies [22, 36, 39, 40, 43], especially support from community leaders such as the chief of police, the mayor, and the health department director (*Like a Good Neighbor theme*). In particular, the initial support of the health department was cited several times throughout the *Point Defiance* NEP case study in Tacoma, Washington [22] as a prominent factor in the successful implementation and ongoing operation of the exchange, and has been critical to the success of the Health Department NEP in this case study. Although both programs in our case study enjoyed broad support from the chiefs of police, support among rank-and-file officers was somewhat divided, which likely contributed to the negative experiences with law enforcement shared by the participants interviewed. To promote police acceptance of the NEP, program leaders of the *LifePoint* NEP involved law enforcement in the planning process, as did both of the West Virginia programs in this case study. Although no participants reported police harassment after the *LifePoint* NEP implementation, the overall attitude of law enforcement with regard to the program remained largely neutral [40]. Notably, community support and overcoming concerns that such programs will encourage continued drug use are not limited to the United States. Programs in remote areas of Thailand experienced similar obstacles to establishing NEPs in response to an HIV problem stemming from injection drug use [44].

Most large urban programs have also described difficulties stemming from the existence of paraphernalia laws and associated policing behavior (*The Legal Conundrum theme)* [38, 39, 41, 43, 45, 46]. The *Point Defiance* NEP did not experience many operational barriers, at least initially, primarily because the chief of police made a conscious decision to not arrest the founder for violation of state paraphernalia laws. Additionally, the state Supreme Court later ruled in favor of the exchange finding that public health interests superseded existing drug paraphernalia laws [22]. However, it is unknown whether or not rank-and-file law enforcement officers also chose to ignore paraphernalia laws in their dealings with PWID, which was a noted barrier reported by participants in our setting. Findings from qualitative interviews in Russia, also suggests the presence of social and structural barriers. More specifically, fear of arrest was identified as a major barrier to PWID NEP attendance in three Russian cities [47].

Funding was a major barrier cited by both West Virginia programs that impacted both current operations and needed expansion plans (*Supply and Demand theme*). A notable exception to this case is the *Point Defiance* NEP in Tacoma, Washington where community support minimized financial barriers. Funding from private citizens, in addition to city and county sources, were cited as contributing factors to a program budget that continued to grow [22]. Similarly, the *LifePoint* NEP case did not report funding difficulties [40]. However, funding difficulties have been reported more recently by NEPs in the United States regardless of geographical location [17].

NEP funding difficulties were exacerbated by exponential growth that necessitated the need for geographic expansion *(Supply and Demand theme)*. This expansion involved delivery of needles to participants who did not or could not attend the original fixed site location, which is similar to the expansion plans of the Free Clinic NEP (i.e., van delivery of supplies to an adjacent county) in this case study, and another study in which NEP participants listed inconvenient location/hours as a barrier to exchange participation [48]. Other urban programs have also cited the benefits from using a mobile versus fixed site program model to reach more PWID [39, 40].

NEP funding has historically been inextricably intertwined with paraphernalia laws in the United States [49]. In a recent analysis of the comprehensiveness of state laws to prevent HCV [50], West Virginia was one of 18 states with the least comprehensive laws. These states had no authorization of statewide syringe exchange, no laws decriminalizing possession and distribution of syringes, and no laws explicitly allowing the retail sales of syringes without a prescription. The absence of explicit direction from the state may have contributed to differing practices among law enforcement officers related to whether or not participants should be legally sanctioned if found to be in possession of syringes, either dirty or clean.

Law enforcement attitudes toward PWID may also impact the approach to drug paraphernalia [51, 52]. Although the law enforcement officials participating in this case study were generally supportive of NEPs, many acknowledged that their fellow officers may share divergent views (i.e., in favor of a criminal approach to drug paraphernalia), and the experiences of NEP participants highlighted this diversity of opinions. This situation is concerning given that recent evidence has suggested a link between syringe sharing and police confiscation of needles, both clean and used. A cross-sectional survey of IDUs in Tijuana and Ciudad Juarez, Mexico observed increased odds of receptive syringe sharing from arrests for possessing both clean needles and used needles [53]. These findings are particularly concerning given the fact that syringe sharing is associated with a 94% increased risk of acquiring HCV [54]. However, the impact of these paraphernalia laws and policing behaviors on the ability of PWID to both bring clean needles back for exchange and have clean needles available for use during injection in rural areas of the United States is currently unknown and warrants further inquiry.

### Limitations

Some study limitations should be noted. It is possible that responses may have been impacted by social desirability bias given the sensitive topic. However, no names or demographic information were collected or reported to help minimize the impact of this bias and increase the validity of the measurement. Additionally, the experiences of other rural programs in both West Virginia and other locales may be somewhat different than the experiences described in this case study given that the small sample size (n = 2 cases) may have precluded the ability to achieve saturation of opinions, views, and themes. However, it is recommended that no more than 5 cases be selected for a qualitative case study, and some methodologists argue that any case beyond 1 can dilute important case details [19]. Furthermore, we did not interview frontline staff that may have further triangulated these results or provided additional barriers and facilitators not mentioned by respondents. Importantly, because we only interviewed current program attendees, the ability to capture important information regarding barriers experienced by PWID who do not regularly attend the exchanges was limited, and represents an area in need of further inquiry. It is also possible that PWID included in our sample may have held a more favorable view of the program given that program directors facilitated access to these participants. Finally, although we were able to reach thematic saturation and triangulate emerging themes with other respondents (i.e., NEP directors, police chiefs), our sample size of 8 PWID across the two NEP cases was small. The perspectives of a larger and more diverse sample of PWID who attend NEPs may corroborate or refute the findings reported here.

## Conclusions

Due to overwhelming need, PWID are often in the dark regarding the precise outcomes that will be experienced during their next injection. Will it be the sought-after high or a disappointing low? Will it be a fatal overdose or a ‘near-miss’? Similarly, the two West Virginia needle exchange programs described in this case study have been forced to take a *shot in the dark* and open NEPs in the absence of model programs, adequate funding, and other resources, due to overwhelming need in their small communities. Despite these challenges, such programs have enjoyed robust community support, and have creatively navigated unexpected problems and challenges to effectively implement needle exchange programs in their communities. However, surging participant volumes amidst ongoing funding challenges coupled with location and transportation barriers make the future trajectory of such programs difficult to predict. Additionally, the efficacy of these programs in preventing transmission of blood borne viruses may be compromised from the legal conundrums created by paraphernalia laws and policing behaviors that may promote needle sharing that is the primary risk for acquiring HCV among PWID. Future studies should investigate these potential barriers to using clean needles in people who inject drugs residing in smaller, less populous areas of the United States.
